# Development of a Survey Tool: Understanding the Patient Experience With Personalized 3D Models in Surgical Patient Education

**DOI:** 10.7759/cureus.35134

**Published:** 2023-02-18

**Authors:** Lauren E Schlegel, Michelle Ho, Kaitlyn Boyd, Robert S Pugliese, Kristy M Shine

**Affiliations:** 1 Otolaryngology, Thomas Jefferson University, Philadelphia, USA; 2 Radiology, Thomas Jefferson University, Philadelphia, USA; 3 Engineering Technology, Drexel University, Philadelphia, USA; 4 Innovation, Thomas Jefferson University, Philadelphia, USA; 5 Emergency Medicine, Thomas Jefferson University Hospital, Philadelphia, USA

**Keywords:** 3d printing, personalized medicine, informed consent, 3d printed models, survey development, patient education

## Abstract

Background: Three-dimensional (3D) printing has been increasingly utilized in the healthcare sector for many applications including guiding surgical procedures, creating medical devices, and producing custom prosthetics. As personalized medicine becomes more accessible and desired, 3D printed models emerge as a potential tool in providing patient-specific education. These personalized 3D models are at the intersection of technological innovation and medical education. Our study group utilized a modified Delphi process to create a comprehensive survey tool assessing patient experience with personalized 3D models in preoperative education.

Methods: A rigorous literature review was conducted of prior patient education survey tools in surgical cases across specialties involving personalized 3D printed models. Through categorization and mapping, a core study team reviewed individual questions, removed duplicates, and edited them into generalizable form. A modified Delphi process was then used to solicit feedback on question clarity and relevance from both 3D printing healthcare experts and patients to create a final survey.

Results: 173 survey questions from the literature were evaluated by the core study team, yielding 31 unique questions for further review. After multiple rounds of feedback, a final survey containing 18 questions was developed.

Conclusion: 3D printed models have the potential to be helpful tools in surgical patient education, and there exists a need to standardize the assessment of patient experience with these models. This survey provides a standardized, generalizable way to investigate the patient experience with personalized 3D-printed models.

## Introduction

Establishing clear communication and understanding between physicians and patients to allow for shared decision making can be difficult in complex medical situations such as informed consent for surgery, discussing treatment plans, or clinical trials. One review found only around 50% of clinical trial patients had an adequate understanding of the elements of informed consent, including the study’s aims, risks, and benefits [1]. More broadly, the Institute of Medicine reports that almost half of American adults - approximately 90 million - have difficulty understanding and utilizing health information [2]. Higher rates of health literacy can lead to better clinical outcomes. This has been demonstrated across a variety of medical conditions including diabetes mellitus, chronic obstructive pulmonary disease, and heart failure [3]. For example, increased health literacy in patients with diabetes is correlated to better glycemic control and poor health literacy in patients with asthma was the strongest predictor of improper use of inhalers [4,5]. Thus, there is a need for strategies to improve the information exchange between physicians and patients.

Currently, physicians largely employ conversation and informational documents to explain health concepts and procedures to patients in the informed consent process, occasionally using visual tools such as sketches, medical imaging, or videos for communication [6-8]. Advances in technology and affordability, along with an increased acceptance by the medical community, have expanded the use of three-dimensional (3D) models in medicine [9]. Compared to the physician experience with custom 3D models for patient care, the patient experience with personalized 3D models is less reported. Published research has reported generally positive experiences utilizing 3D models for patient education and informed consent processes. For example, within the field of urology, patient-specific 3D models have been shown to increase patient understanding of anatomy, disease, and procedures [10]. Similar types of models have been used in preoperative patient consultations for neurosurgical, otolaryngologic, and orthopedic procedures with positive results [11-13].

Across these studies, researchers used a variety of measures to evaluate patients’ experiences with personalized 3D models ranging from subjective measures of satisfaction and understanding of the diagnosis to objective measures, such as anatomical knowledge and surgical approach questions. Because there exists no standardized set of questions to assess patient experiences with 3D models in pre-surgical information exchange, it is difficult to compare overall utility across specialties. Moreover, existing surveys are often highly medicalized, specialty-specific, and only readable at an advanced level. The majority do not involve lay individuals (i.e. patients) in the process to ensure their understanding and priorities are reflected.

The purpose of this research was to collect the prior body of survey questions presented to patients exposed to 3D printing in surgical applications as a basis for developing a standardized, easily understood tool. By utilizing a modified Delphi method, the survey went through rounds of anonymous feedback from patients and clinicians. This survey can be utilized to measure the benefit of patient-specific 3D printed models in patient education around surgical planning that reflects both patient and expert feedback.

## Materials and methods

Study team

The core study team consisted of five individuals including a physician, physician-scientist, social scientist, engineer/medical student, and 3D printing lab manager. The 3D printing expert team consisted of nine individuals with previous experience working with personalized 3D printed models as part of clinical and/or surgical care at the study team’s institution. The patient team consisted of 18 non-medical individuals who had previous experience with a medical procedure. Participants were recruited through email as a convenience sample. This study was reviewed and approved by the Thomas Jefferson University Institutional Review Board (approval #20G.044). 

Literature search 

A literature search was conducted using the electronic databases Pubmed, Ovid, and SCOPUS. The following keywords were searched in the title and abstract: model OR planning OR training OR education OR teaching OR assessment OR skills OR simulation AND "3D print" OR "3D printing" OR "3D Printed" OR "three-dimensional print" OR "three-dimensional printing" OR "three-dimensional printed". Articles were screened by reviewing title and abstract to determine if they met inclusion criteria and warranted full-text review. Inclusion criteria required the article be 1) published in the English language, 2) a primary article, 3) use patient-specific 3D models for patient education, and 4) administer questions assessing patient experience with 3D printed models. Articles that initially passed inclusion criteria underwent data extraction. 

Data extraction

Articles were examined in a systematic way and data extraction included study year, specialty, total questions administered, use of objective and subjective questions, and type of rating scale used. When available, full-text questions from surveys were extracted. 

Categorization 

To understand the scope of questions being asked in prior studies and create a shorter generalizable survey, the full-text questions were assigned to one of six categories - anatomy, communication, complication, diagnosis, experience, procedure, visualization - based on the focus of the question, as determined by the core study team through consensus (Table 1). Based on matching categories, similar questions were grouped and merged into one representative question. All questions, both unique and representative merged questions, were modified by two study team members to remove references to specific diagnoses or procedures resulting in a general, specialty-independent form. 

**Table 1:** Categorization Example Example of the categorization process in which each question was assigned a category and similar questions were merged and modified to a simple generic form. 2D, two-dimensional; 3D, three-dimensional; CT, computerized tomography

Study	Original question	Generic question		Category				Generic Question	
Komai 2016 [14]	Compared to 2D or 3D CT imaging, did the 3D model help understand the risks of operation-related complications such as bleeding and urinary leakage	Compared to 2D or 3D CT imaging, did the 3D model help understand the risks of operation-related complications	Complication		Merged		The model was useful for enhancing knowledge of possible complications		
Yoon 2019 [15]	I understand that potential complications includes death, and hospital stay can be lengthened.	I understand the potential complications	Complication						
Alshomer 2019 [16]	The information presented gave a clear vision about the risks and complications of the intervention	The information presented gave a clear vision about the risks and complications of the intervention	Complication						
Klosterm-an 2018 [17]	In the future I would recommend it [3D model] to family and friends	In the future I would recommend it [3D model] to family and friends	Experience		Merged		I would recommend a 3D model to others		
Bizotto 2016 [18]	Would you suggest to other patients to request a 3D printed model of their fracture before the surgery?	Would you suggest to other patients to request a 3D printed model of their diagnosis before the surgery?	Experience						

Mapping 

**Figure 1:**
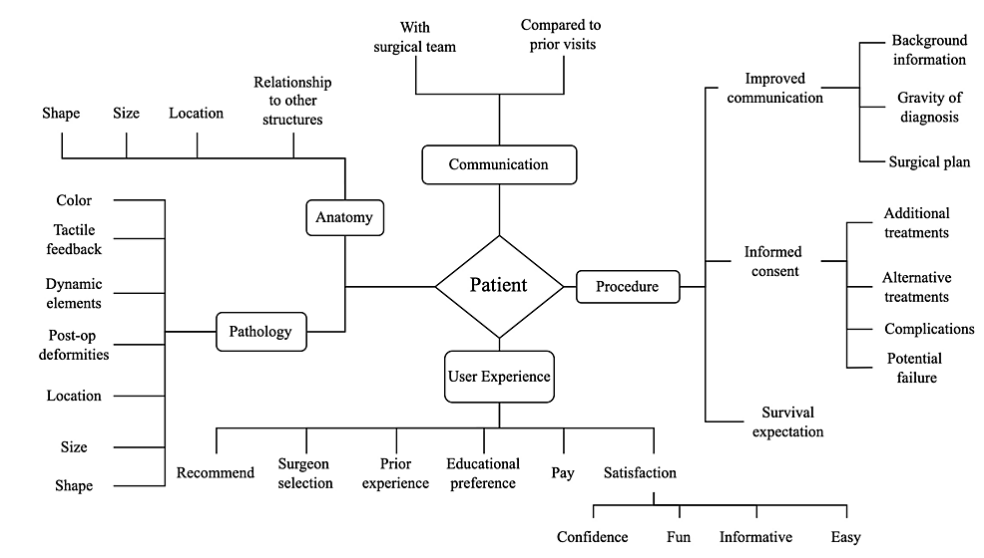
Diagram of Mapping Process

To further condense the survey to a manageable length, the core study team reviewed all questions remaining after the categorization and generalization process to determine dominant themes (i.e. anatomy/pathology, communication, procedure, and user experience) and grouped the questions accordingly (Figure 1). Questions that were considered too narrow in focus to apply broadly across specialties/procedures as determined by consensus were removed. Each endpoint of the diagram was converted into a short, generalizable question. The core study team reviewed these questions in the context of known features and clinical use of patient-specific 3D models and considered survey question supplementation if indicated. 

Delphi process 

Round One

A modified Delphi process was used to review the list of questions generated post mapping (Appendices). For the first round of feedback, the survey was sent to a team of healthcare-associated 3D printing experts. For each question, anonymous feedback on question clarity and relevance was obtained and experts noted if it should be retained as-is, reviewed, or removed. Clarity and relevance were graded using a 5-point Likert scale and retention on a 3-point scale (3- retained as-is, 2- reviewed, or 1- removed). Questions that received < 1.5 (i.e. scored more toward removal) on retention were automatically removed. Questions that received ≥ 3.0 (i.e. neutral) for relevance and clarity were kept for subsequent feedback rounds either as written, or reworded/combined with another similar question based on reviewer comments by at least two members of the core study team through consensus discussion. All other questions not meeting the above criteria underwent review by at least two members of the core study team and these questions were either reworded or combined with another similar question for inclusion in a subsequent feedback round or removed from further consideration based on reviewer comments (Table 2).

**Table 2:** Example Evolution of Question Through Delphi Process

Original survey question	The model improved my confidence (Strongly disagree = 1, Disagree = 2, Neutral = 3, Agree = 4, Strongly agree = 5)
Feedback questions	This question is clear (Strongly disagree = 1, Disagree = 2, Neutral = 3, Agree = 4, Strongly agree = 5) This question is relevant (Strongly disagree = 1, Disagree = 2, Neutral = 3, Agree = 4, Strongly agree = 5) This question should be… (Retained as-is, Reviewed, Removed) Comments
Feedback results	This question is clear – 66% of experts strongly agreed the question was clear (average = 4.75) This question is relevant – 25% of experts strongly agreed the question was relevant (average = 3.5) This question should be … - 50% of the experts believed the question should be retained as-is (retention score = 2.5)
Feedback comments	“improved my confidence in what?” “confidence in decision making”
Outcome	Original survey question edited to clarify keywords and address comments: The model improved my confidence in medical decisions.

Round Two

**Figure 2:**
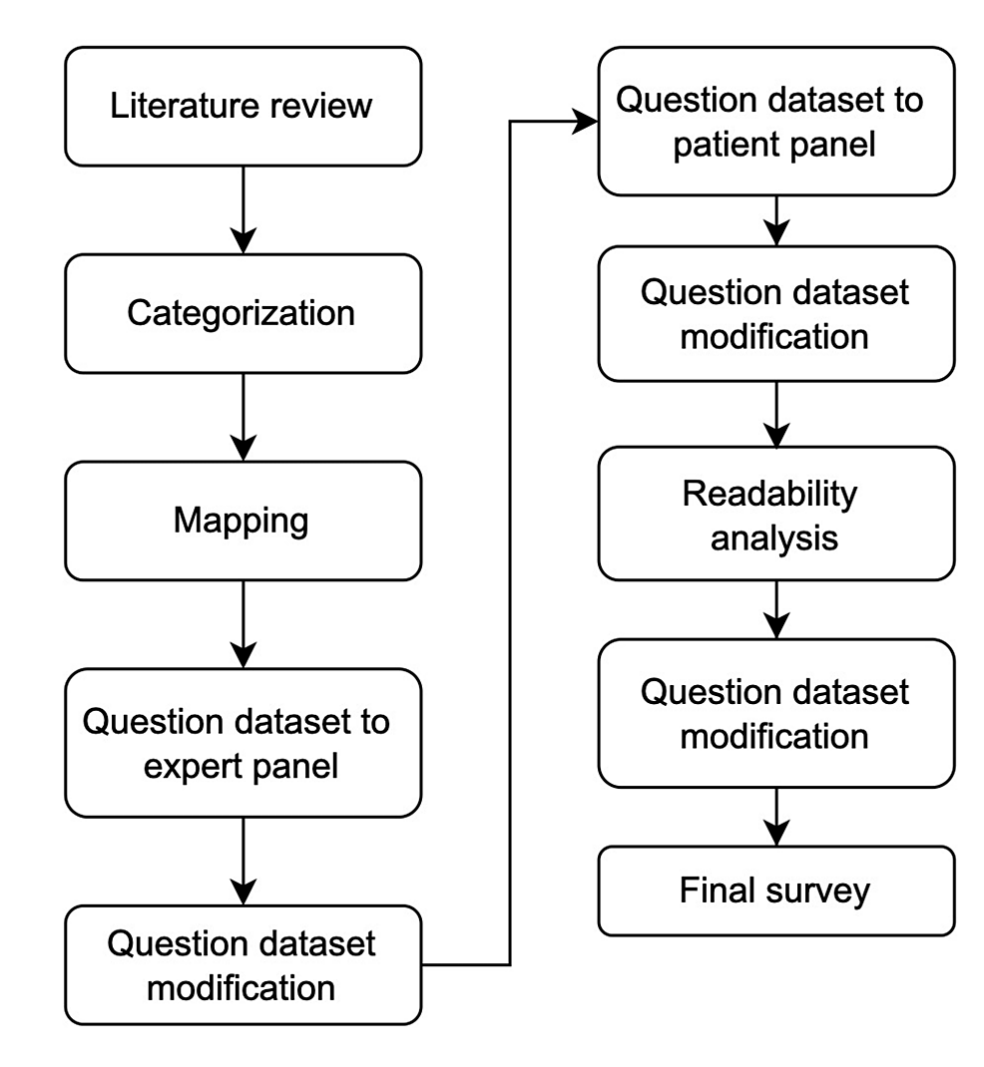
Flow Diagram of Modified Delphi Process

In the next round of feedback, a group of patients reviewed the list of questions generated from the expert review (Appendices). In a similar anonymous fashion, the patients were asked to review each question for relevancy, clarity, and retention. All questions underwent the same process as before based on the retention, relevance, and clarity scores. The resultant patient survey, both instructions written by the study team and individual questions, were evaluated for readability using the Flesch-Kincaid grade level [19]. Figure 2 displays the entire modified Delphi process for developing the survey. The instructions and questions were then modified to ensure readability at an 8th-grade reading level. Length of survey was calculated to ensure appropriate brevity of under five minutes [20].

## Results

Literature search

A total of 7141 articles were found on initial inquiry. After removing duplicates, 3705 records remained and underwent title and abstract face validity screening based on inclusion criteria. Sixty-three full text articles were selected for full content review; however upon complete review, only 30 of the 63 articles met inclusion criteria. Eight specialties were represented through these articles with orthopedic surgery being the most frequent specialty represented (n=9), followed by urology (n=6), neurosurgery (n=5), general surgery (n=3), cardiology (n=3), plastic surgery (n=2), otolaryngology (n=1), and obstetrics and gynecology (n=1).

Categorization and mapping results

A total of 173 full-text survey questions were extracted from articles. These questions were split into categories based on general focus including diagnosis (n=47), procedure (n=43), anatomy (n=24), communication (n=22), experience (n=22), complications (n=13), and visualization (n=2). Following merging of similar/duplicate questions, 53 unique generic questions were generated (13 anatomy, 4 communication, 2 complication, 7 diagnosis, 15 experience, 11 procedure, and 1 visualization). Using the process of mapping questions to dominant themes, 31 key themes were identified and a unique question for each was generated from the questions in that group, yielding 31 survey questions (11 anatomy, 2 communication, 8 procedure, 10 experience).

Modified Delphi process

Ten healthcare-associated 3D printing experts submitted anonymous feedback on the questions. Of the 11 anatomy questions, two questions were removed due to low retention scores (mean < 1.5). One question received an average score of ≥ 3.0 for relevancy and clarity and was retained but reworded based on expert feedback. Based on review of the remaining question for relevance and clarity scores and discussing expert comments, only one other question was retained after rewording.

Of the communication questions, one question received an average score of ≥ 3.0 for relevancy and clarity and was retained as written. The second question received a score of ≥ 3.0 for relevancy but was below the cutoff for clarity so it was retained but reworded.

A total of eight questions relating to procedure were reviewed by the experts. Three received an average score of ≥ 3.0 for relevancy and clarity and were retained but reworded based on expert feedback. Of the remaining procedure questions, two pairs of questions were found to be very similar and were retained but combined based on expert feedback. The final question was also retained but reworded for clarity.

Ten questions relating to user experience with the patient-specific 3D models were reviewed by the experts and all 10 received an average score of ≥ 3.0 for relevance and clarity and were retained. One was kept as-is, two were combined/reworded, and seven were reworded for clarity. At the end of this round of expert feedback, the survey contained 18 questions (2 anatomy, 2 communication, 6 procedure, 8 experience). 

In the second round of the Delphi process, 18 patients submitted feedback for the survey. There were no questions that scored below a 1.5 for retention and therefore none were removed. All questions scored above 3.0 for both relevance and clarity and were retained but underwent review by at least two members of the core study team for clarity using qualitative patient feedback as a guide. Seven questions were retained as-is and 11 questions were reworded for clarity/readability. The final survey therefore contained 18 questions (3 anatomy, 2 communication, 6 procedure, 6 experience, 1 comment) with a Flesch Kincaid Grade Level of 7.8, indicating a below 8th-grade comprehension level (Table 3). 

**Table 3:** Final Questionnaire 3D, three-dimensional

Anatomy	
	The model was easy to understand.
	The model’s colors helped me identify the affected part of my body.
	The model’s scale (being life-sized) helped me understand the affected part of my body.
Communication	
	The model helped me communicate with my doctor.
	Compared to visits with no model, having the model at my appointment improved the experience.
Procedure	
	The model helped me understand my medical diagnosis.
	The model helped me understand the likely progression of my condition.
	The model helped me understand my treatment options.
	The model helped me understand the doctor’s plan.
	The model helped me understand possible risks during my procedure.
	The model helped me understand possible complications after my procedure.
Experience	
	The model improved my confidence in making medical decisions.
	I want my doctor to use 3D models in my future care.
	I am more likely to choose a doctor who uses 3D models compared to one who does not.
	I would recommend the use of similar 3D models to other patients.
	I would be willing to pay for a personalized 3D model.
	Overall, I liked having a 3D model used as part of care.
	Comment: Do you have any comments about having a 3D model used in your care?

## Discussion

The strength of this research comes from the multi-faceted approach used to generate a specialty-agnostic, comprehensive set of survey questions that can be presented to patients. Importantly, we chose the landscape of prior survey-based research studies in orthopedic surgery, otolaryngology, general surgery, urology, and others as the foundation for our work. The questions on which our survey is based stem from the prior work of 30 other research groups across nine specialties, allowing us to take into consideration the values of each specialty.

From the beginning, we prioritized creating a high-quality survey that takes into account aspects such as sentence and survey length in addition to accessible vocabulary. Given the known issue of survey fatigue and its contributing factors including survey length, topic, and question complexity, we aimed to develop a survey with less than 20 short, concise questions [21]. To condense the 173 questions that were initially extracted, questions were split into categories based on the focus of the question and similar questions were merged. To condense them further, a round of mapping overarching themes yielded 31 questions. By extracting the most basic contents of each question, a generic but comprehensive survey could be derived from complex specialty-specific questions.

The Delphi method is a natural way to integrate the perspectives and values of both 3D printing experts and patients in an anonymous way into the survey [22]. In the category of anatomy, the healthcare-associated 3D printing experts found many questions to be too technical, related to the accuracy of the model and its relationship to other structures. These are common ways of thinking about anatomy in the healthcare field, but based on clinician experience with explaining medical procedures, felt to be difficult and less relevant. 3D printing experts’ feedback guided questions to be reworded when necessary and removed if no longer deemed relevant, thereby creating a more patient-centered survey.

Patient feedback provided valuable insight for rephrasing questions and was overwhelmingly positive with most questions rated highly in both relevance and clarity. The study team found it critical to include patient perspectives while developing the questionnaire to empower patients to promote the aspects of their pre-surgical educational experience that they value most. The predominance of procedure and experience-related questions in the final survey may reflect the aspects of patient education that these personalized 3D models can address. Asking questions about patient experience may be the first step in learning about the value that patients place on having this educational tool as part of their preoperative appointment. Of course, the patient's experience with the model will be influenced by the educator using the 3D model. Additionally, the complexity of the anatomy or the time the physician spends explaining it could influence the model's ability to help patients understand. Therefore it is important to do this work to determine if patients find these personalized 3D models helpful and what they learn from the model. 

A well-known barrier to effective physician-patient communication is the use of language and reading levels that are too complex [23]. While the average American reads between a 7th- and 8th-grade level, educational tools like 3D models do not rely heavily on language alone [24,25]. Despite recommendations that patient health information should be delivered at a low readability level, many studies have shown the healthcare system has not done so yet [26]. The use of visual aids, like personalized 3D models, holds the potential to provide an alternative way to explain difficult concepts to patients.

To our knowledge, this is the first study to describe a methodology for developing a comprehensive survey tool to assess patient perspectives on the use of 3D models in physician-patient communications for surgical applications. While previous researchers have captured patient feedback on 3D models, such feedback stems from independently generated surveys relevant to their specific specialties and surgical procedures, making it highly variable and difficult to compare across applications. Thus, a complete picture of the value of such models remains out of reach. The survey is now being utilized at our institution across specialties. In the future, the study team plans to validate the use of this survey and review the responses to determine the utility of 3D personalized models in addressing the communication barrier between clinicians and patients.

The use of the Delphi process incorporated both 3D printing expert and patient feedback to better reflect the values of patient education researchers and the opinions and needs of patients. This survey can be used to gather data to improve the patient experience using personalized 3D models. Additionally, by utilizing this survey in a standardized fashion, we may determine that some model types or those used in specific specialties have more value than others. 

Our study did have a few limitations. Because patients involved in survey development were recruited through email as a convenience sample, selection bias may be an issue. In addition, due to the coronavirus pandemic they were unable to hold a model while giving their feedback. To fill this deficit, numerous pictures of 3D models that have been used in pre-surgical education were shown to the non-medical participants prior to collecting their feedback. Due to our decision to utilize both clinician and patient panels for feedback, we gained rounds of anonymous feedback from multiple shareholders. This meant the same group of people did not get the opportunity to evaluate the survey more than once. 

## Conclusions

Personalized 3D-printed models have the potential to overcome communication barriers by providing a visual tool to allow for better patient education. The purpose of this study was to use a strong base of previously published literature in combination with a modified Delphi process to work with multiple stakeholders to develop a broadly applicable survey to measure the potential of personalized 3D models in patient education. Taking into account years of literature across many specialties ensures the longevity and generalizability of this survey, providing valuable data on this tool in patient education.

## Appendices

**Table 4:** Questions Posed to Expert Panel

Anatomy
The model’s size is accurate.
The model’s location is accurate.
The model’s shape is accurate.
The model’s relationship to other structures is accurate.
The model’s region of interest location is accurate.
The model’s region of interest size is accurate.
The model’s region of interest shape is accurate.
The model’s region of interest color is accurate.
The model’s region of interest tactile feedback is accurate.
The model’s region of interest interactive features are accurate.
The model’s post procedure shape is accurate.
Communication
The model was useful for communicating with my clinician.
The model was useful compared to prior visits.
Procedure
The model was useful for survival expectation.
The model was useful for enhancing knowledge of alternative treatment plans.
The model was useful for enhancing knowledge of the possibility of additional treatments.
The model was useful for enhancing knowledge of possible complications.
The model was useful for understanding the procedure may fail.
The model was useful for communicating the clinician’s plan.
The model was useful for communicating the gravity of diagnosis.
The model was useful for background knowledge.
Experience
I would want a 3D model for future procedures.
I would recommend a 3D model to others.
I would prefer my clinician use a 3D model in the future.
Use of a 3D model would influence my choice of clinician in the future.
I would pay for a 3D model in the future.
I have prior experience with 3D models.
The model was fun.
The model was informative.
The model was easy to use.
The model improved my confidence.

**Table 5:** Questions Posed to Patient Panel

Anatomy
Having the model be life-sized helped me understand the problem with my body.
The model’s colors helped me identify parts of my body.
Communication
The model was useful for communicating with my doctor.
The model enhanced my appointment with my surgeon, compared to other visits with no model.
Procedure
The model was helpful in understanding the risks associated with my procedure.
The model was useful for understanding other possible treatments.
The model was useful for understanding potential complications of the procedure.
The model helped me to understand the doctor’s plan.
The model was useful for understanding the seriousness of my diagnosis.
The model was useful for understanding the progression of my condition.
Experience
I want my doctor to use 3D models in my future care.
I would recommend similar use of 3D models to other patients.
I am more likely to choose a doctor who uses 3D models compared to one who does not.
I would pay for a personalized 3D model.
I liked having a 3D model used as part of my care.
The model helped me understand my medical problem.
The model was easy to understand.
The model improved my confidence in medical decisions.
Comment: Do you have any comments about having a 3D model used in your care?
